# Streptococcus pneumoniae-Related Infective Endocarditis Complicated by Development of Psoas and Epidural Abscesses

**DOI:** 10.7759/cureus.78691

**Published:** 2025-02-07

**Authors:** Fatemeh Jafari Roshan-Zamir, Shantell Ceaser, Kristy Chin, David Mas

**Affiliations:** 1 Neurology, William Carey University College of Osteopathic Medicine, Hattiesburg, USA; 2 Internal Medicine, Ochsner University Hospital and Clinics, Lafayette, USA; 3 Internal Medicine, Louisiana State University Health Sciences Center at Ochsner University Hospital and Clinics, Lafayette, USA; 4 Family Medicine, Louisiana State University Health Sciences Center at Ochsner University Hospital and Clinics, Lafayette, USA

**Keywords:** case report, epidural abscess, infective endocarditis, mitral valve vegetation, neurology, streptococcus pneumoniae

## Abstract

*Streptococcus pneumoniae *(*S. pneumoniae*), a gram-positive bacterium in the upper respiratory tract, can cause pneumonia, meningitis, and bloodstream infections. *S. pneumoniae* septicemia may lead to cardiac valve seeding, increasing the risk of epidural and psoas abscesses. Understanding its pathology is crucial for improving early detection and intervention.

A 55-year-old male patient presented with fever, acute disorientation, and confusion. Initial broad-spectrum antibiotics were adjusted following positive blood cultures for *S. pneumoniae*. While meningitis was the primary concern, further imaging revealed mitral valve vegetation, an epidural abscess (L2-L4), osteomyelitis, and a psoas abscess. Due to an unsuccessful lumbar puncture, CSF analysis was not obtained. The patient received IV Ceftriaxone and Vancomycin and underwent a successful laminectomy, completing six weeks of antibiotics.

This case highlights the severe complications of *S. pneumoniae* infective endocarditis, particularly its hematogenous spread leading to an epidural abscess. While cardiac valve seeding is well-documented, its role in epidural abscess formation is often overlooked. Given the diagnostic challenges of spinal epidural abscesses due to their resemblance to meningitis, accurate and timely diagnostics are critical for optimizing treatment and preventing deterioration.

## Introduction

*Streptococcus pneumoniae* (*S. pneumoniae*), commonly known as pneumococcus, is primarily associated with pneumonia but can also cause severe systemic infections, including meningitis, bacteremia, and infective endocarditis. If left untreated, these infections may progress to secondary complications such as spinal epidural and psoas abscesses. As de Leau et al. have noted, pneumococcal bacteremia can lead to meningitis, peritonitis, and endocarditis [1], with the potential for hematogenous spread to the central nervous system. Leavitt et al. described the classic triad of spinal epidural abscess (SEA)--fever, back pain, and neurological deficits--though its presentation can be variable and mimic other infections [2].

We present a case of a 55-year-old male who arrived at the emergency department with fever and acute disorientation, leading to the incidental discovery of mitral and aortic valve vegetation. Further imaging revealed an epidural abscess, osteomyelitis, and a psoas abscess, emphasizing the rare but severe sequelae of *S. pneumoniae* infective endocarditis. Despite its recognized role in cardiac valve seeding, its contribution to epidural abscess formation remains underreported.

This case expands on the existing literature by demonstrating *S. pneumoniae's* capacity for extensive hematogenous dissemination beyond typical sites of infection. Given the diagnostic challenges of SEA, early recognition and targeted interventions are crucial to preventing delays in treatment. Publishing this case underscores the importance of maintaining a high index of suspicion for atypical complications of pneumococcal bacteremia, ultimately aiding clinicians in improving diagnostic accuracy and patient outcomes.

## Case presentation

A 55-year-old male with a past medical history of hypertension, hyperlipidemia, syphilis, and schizophrenia presented with fever, acute confusion, and disorientation. He had sought medical attention at the emergency department (ED) four days prior, reporting myalgias, chills, diaphoresis, neck pain, and back pain. He was diagnosed with influenza and started on Tamiflu. During his most recent ED visit, neurological examination revealed disorientation, an anxious demeanor, delayed speech, impaired memory and concentration, cervical rigidity, and a positive Kernig’s sign. 

Laboratory tests showed leukocytosis with a white blood cell (WBC) count of 19,000/µL (reference range: 4,000-11,000/µL), microcytic anemia, hyponatremia, elevated brain natriuretic peptide, and an increased lactic acid level of 2.9 mmol/L (reference range: 0.5-2.2 mmol/L). Blood cultures during admission detected *S. pneumoniae*, for which 2g IV ceftriaxone q 12 hours was initiated. The medical staff attempted to perform a lumbar puncture, but despite their efforts were unable to obtain cerebrospinal fluid for analysis. Treatment with a combination of vancomycin and Zosyn was initiated thereafter. Computed tomography (CT) scans of the head, cervical, and lumbar spine without contrast showed no evidence of infection. 

**Figure 1:**
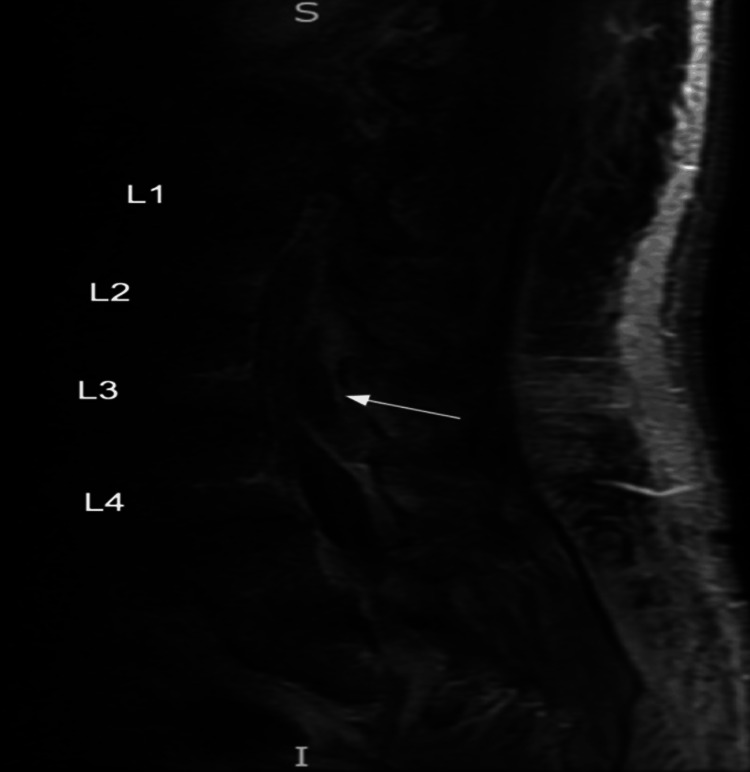
MRI imaging of an epidural abscess (white arrow) Magnetic resonance imaging (MRI) sequence T2 with gadolinium contrast of the lumbar spine exhibits a T2 hyperintense rim-enhancing collection in the dorsal epidural space at the L2-L4 level, with a thickness of 8mm. This results in the anterior displacement of the cauda equina nerve roots, accompanied by a partial collapse of the thecal sac.

**Figure 2:**
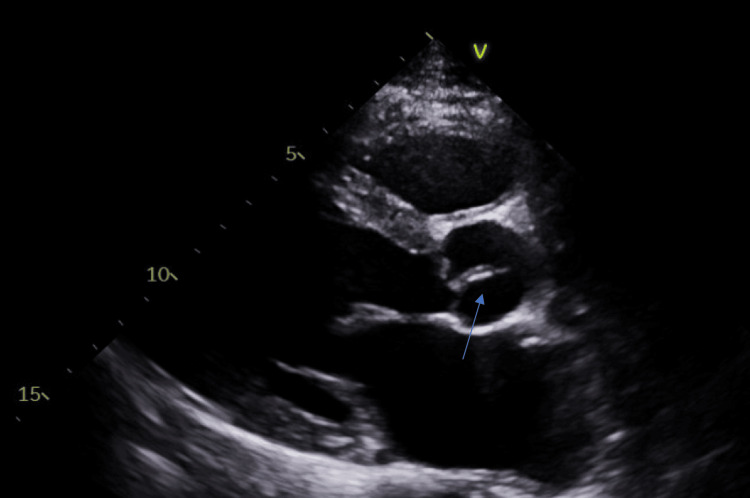
Mobile mass in the posterior valve of mitral valve (blue arrow) Image displaying a sizable mobile echogenic pedunculated mass is observed on the posterior leaflet of the mitral valve, accompanied by a small vegetation on the aortic valve in the transthoracic echocardiogram (TTE).

An MRI of the lumbar spine with contrast detected a dorsal epidural abscess at L2-L4, L4-L5 right osteomyelitis, and a small right psoas muscle abscess (Figure 1). The patient was diagnosed with an epidural abscess secondary to infective endocarditis and received 2g IV ceftriaxone and 1.75 g IV vancomycin for a therapeutic duration of eight weeks. Further evaluation using a transthoracic echocardiogram (TTE) revealed an echogenic mass on the posterior leaflet of the mitral valve, accompanied by moderate atrial valve regurgitation (Figure 2). Due to persistent leukocytosis and the presence of an epidural abscess, the patient was transferred to an outside hospital for a laminectomy and washout. Post-surgery, he was discharged home with home health services and continued the remaining course of antibiotics. This case highlights the potential consequence of hematogenous seeding from *S. pneumoniae-related* infective endocarditis, leading to significant bacteremia and the development of an epidural abscess.

## Discussion

After identifying *S. pneumoniae*-induced mitral-valve seeding as the origin of the epidural abscess, a precise treatment plan was devised for the patient. Given the clinical similarity between SEA and meningitis, as mentioned in Table 1, the ED administered vancomycin and piperacillin/tazobactam, along with dexamethasone. During the seven-day period following admission, the patient's condition remained undiagnosed, and no MRI was conducted at that time, as the initial concern was meningitis rather than an SEA. Throughout this time, the individual continued to suffer from persistent bilateral lower back pain, accompanied by discomfort when extending both legs and knees. The treatment plan during this period involved administering 2g IV ceftriaxone every 12 hours to cover empiric meningitis, latent syphilis, and endocarditis. 

**Table 1:** Spinal epidural abscess The table provides an overview of spinal epidural abscesses, listing key clinical features such as back pain, fever, and neurological deficits. It also highlights common risk factors, including diabetes and intravenous drug use, while outlining the standard management approach, including antibiotic therapy and potential surgical intervention.

Clinical features	Clinical triad: fever, back pain, neurological deficits
Risk factors	Immunocompromised, endocarditis, alcoholism, intravenous drug use, steroid therapy, diabetes, trauma
Diagnostic approach	MRI of spine with contrast echo of cardiac valves
Treatment	Antibiotics: empiric coverage for staphylococcus aureus (vancomycin 1.75 mg IV Q12H), neurosurgical intervention

On the seventh day of admission, an MRI of the lumbar spine with contrast raised concerns for a dorsal epidural abscess at L2-L4, accompanied by L4-L5 osteomyelitis and a right psoas abscess. Consequently, the patient was transferred to an external facility for a laminectomy and washout. Following the surgical procedure, the patient continued ceftriaxone for an additional two weeks, resulting in an overall eight-week treatment duration. Upon discharge, a peripherally inserted central catheter (PICC) line was inserted to facilitate ongoing antibiotic treatment.

SEA can manifest through various routes in the human body, with hematogenous spread, extension from infected tissues, and direct inoculation being the primary mechanisms [3]. Notably, SEAs can arise as secondary complications of conditions such as sepsis-related infective endocarditis, dental abscesses, and intravenous drug use. *Staphylococcus aureus* is the predominant pathogen associated with SEA, whereas *Streptococcus pneumoniae* and *Escherichia coli* represent rare manifestations [1] [1]. In this case, the patient developed infective endocarditis with concurrent bacteremia caused by *S. pneumoniae*, which disseminated hematogenously to invade the heart and damage the cardiac valves, ultimately spreading to the epidural space and causing an SEA.

The manifestation of a SEA may initially exhibit non-specific symptoms, but the classic triad comprising spinal pain, fever, and neurological dysfunction is indicative of its presence [2]. The similarity of these symptoms to bacterial meningitis can potentially impede prompt treatment initiation. Certain warning signs, particularly intense pain in the lower back coupled with difficulty extending the legs (Kernig’s sign), should raise suspicion of potential infections in the lumbar area. To ensure accurate diagnosis and optimal treatment, it's crucial to conduct a thorough patient history review alongside prompt and appropriate diagnostic testing. This comprehensive approach forms the foundation for effective medical care and decision-making. Neurological symptoms such as altered mental status, motor weakness, nerve pain, and bowel or bladder dysfunction, play a crucial role in differentiating SEA from meningitis [2]. The development of an abscess within the epidural space can exert significant pressure on nerve roots, resulting in shooting pain. Inflammation in the affected area may manifest as redness, heat, and tenderness upon palpation. Clinicians should be vigilant in considering these clinical indicators to ensure accurate and timely diagnosis, facilitating effective management strategies.

When a SEA is suspected, obtaining appropriate imaging becomes imperative to determine the location and depth of abscess formation. While an epidural abscess is a potential outcome, the formation of a psoas abscess or lumbar spine osteomyelitis should also be considered. The gold standard for SEA diagnosis is an MRI with gadolinium, demonstrating a sensitivity exceeding 90% [4]. The patient's lumbar MRI was not performed until the seventh day of hospitalization as the initial presentation in the emergency department mimicked symptoms of meningitis. This delay resulted from three unsuccessful lumbar puncture attempts, further complicating the diagnostic process. On MRI spine with gadolinium contrast, an epidural abscess may manifest as either homogenous enhancement indicative of granulomatous thickened tissue or a liquid abscess surrounded by inflammatory tissue, displaying enhancement with gadolinium [5]. Such imaging techniques with an emphasis on rapid turnaround can greatly enhance the precision and speed of diagnosis, ultimately informing and improving patient care.

## Conclusions

This case highlights the rare but severe complications of *S. pneumoniae* infection, particularly its progression from infective endocarditis to SEA, a potentially life-threatening condition. The hematogenous spread of *S. pneumoniae* led to multisystem involvement, affecting both the cardiac and spinal regions. This necessitated a swift, comprehensive approach to diagnosis and treatment to prevent irreversible damage, including neurological deficits and potential long-term disability. Despite initial diagnostic challenges due to the overlapping symptoms with bacterial meningitis, which can include fever, confusion, and neurological deficits, timely MRI imaging and subsequent surgical intervention led to a positive outcome.

The case emphasizes the critical importance of early recognition and a thorough diagnostic workup, especially in patients with atypical symptoms that may obscure the presence of more serious conditions like SEA. Atypical symptoms in this context might include vague or nonspecific complaints such as neck or back pain, fatigue, or mild cognitive disturbances, which can be mistakenly attributed to less severe conditions. These symptoms, when coupled with a history of recent infections or bacteremia, should raise suspicion for more complex, multisystem involvement.

Rapid imaging, in combination with aggressive antimicrobial therapy and timely surgical intervention, is essential to mitigate the devastating consequences of *S. pneumoniae *infective endocarditis and its complications. Recognizing and addressing these rare but critical complications can significantly enhance patient outcomes by ensuring that treatment is initiated without delay, improving both survival rates and recovery prospects.
